# Potential prognostic roles of serum lactate and Creatine kinase levels in poisoned patients

**DOI:** 10.1186/s12873-020-00326-x

**Published:** 2020-04-29

**Authors:** Alireza Golaghaei, Hossein Hassanian-Moghaddam, Shahin Shadnia, Nasim Zamani, Fatemeh Amraei

**Affiliations:** 1 AJA University of Medical Sciences, Tehran, Iran; 2 Department of Clinical Toxicology, Loghman Hakim Hospital, Shahid Beheshti University of Medical Sciences, Tehran, Iran; 3 Social Determinants of Health Research Center, Shahid Beheshti University of Medical Sciences, Tehran, Iran; 4 Toxicological Research Center, Shahid Beheshti University of Medical Sciences, Tehran, Iran; 5 Tehran University of Medical Science, Tehran, Iran

**Keywords:** Lactate, Creatine kinase, Poisoning, Prognostic factor

## Abstract

**Background:**

Examination of serum lactate level and its changes, as an indicator of tissue oxygenation, as well as level of creatine kinase (CK) inhibitors, as a factor of mortality which partially expresses heart, brain, and muscle damage, may be considered as tools to determine prognosis in critically ill patients. We aimed to evaluate these two factors as potential prognostic factors in critically poisoned patients admitted to our toxicology ICU.

**Method:**

This is a cross-sectional descriptive-analytic study that was performed on poisoned patients referred to emergency department of Loghman Hakim Hospital. One-hundred critically poisoned patients who had been admitted to ICU were conveniently chosen using a random number table and included into the study after obtaining consent forms from their next of kin. Their serum lactate and CK levels were checked on admission. These levels were compared subsequently between survivors and non-survivors to seek for their potential prognostic role.

**Results:**

In a total of 100 patients enrolled, 61 were male. Serum level of lactate (with a cut off of 26 mg/dL) and serum CK with a cutoff point of 169 U/L could have prognosticated death with sensitivity and specificity of 78 and 77% (for lactate) and 74 and 62% (for serum CK), respectively.

**Conclusions:**

In poisoned patients, serum lactate and CK can be used as possible prognostic factors because they rapidly increase in the serum and are easily detectable.

## Background

Determination of the poisoned patients’ prognosis since admission is an important factor that should always be considered by clinical toxicologists because of the limited intensive care unit (ICU) beds and nurses [1, 2].

In Iran, almost 650 people die annually due to poisoning and its complications. Although the pattern and trend of poisoning is different all over this country, the most common drugs causing poisoning include sedatives, acetaminophen, and cardiologic drugs [3]. This is while there is limited access to professional health care personnel and facilities for intoxicated patients and this emphasizes the fact that these patients need to be prognostically evaluated since admission in order that those who would best benefit from specific care can be prioritized [4]. Although mortality of poisoning depends on the type of poisoning, early diagnosis and authentic treatment can be lifesaving [3].

Serum levels of lactate and creatine kinase (CK) have been used as possible prognostic factors in critical patients [5, 6], although studies on poisoned patients are not sufficient. Serum lactate is a potentially useful biomarker to risk-stratify patients with severe sepsis. However, it is plausible that elevated serum lactate is simply a manifestation of clinically apparent organ dysfunction and/or shock [6]. On the other side, serum CK can represent muscle, heart, and brain injuries. Measuring serum level of this enzyme is playing an important role in determination of severity of toxicity in patient at the risk of vital organs damage [7].

Since data in this regard is lacking in poisoned patients, we aimed to evaluate the potential role of serum lactate and CK as possible prognostic factors in 100 critically poisoned patients referred to a clinical toxicology tertiary center who were admitted to toxicology ICU due to severe poisoning.

## Methods

In a cross-sectional descriptive study, ***all*** patients who referred to poisoning emergency department of Loghman Hakim hospital between January 2016 and December 2017 were considered to be enrolled into the study. They were from those with confirmed poisoning and need to be admitted to ICU due to severity of poisoning (need for intubation, invasive treatments, and maintenance of antidotes) [2]. On the other hand, those whose treatment had begun before admission (referred from other centers), poisonings with critically increasing levels of lactate as a nature of it (including biguanids and anticonvulsants), and those whose next of kin did not consent to be included, were excluded from the study.

One-hundred critically ill patients admitted to toxicology ICU were conveniently chosen and enrolled into the study using a random number table. Blood samples were taken and serum lactate and CK levels were determined using spectrophotometry in the laboratory. A questionnaire which was designed by our team was used to collect the patients’ data. Information included patients’ demographic characteristics, on admission vital signs and lab tests, primary CK and lactate, final diagnosis, hospital stay, and final outcome which was recorded for every single patient.

Statistical analysis was carried out using statistical package for social software version 25. Mean (SD) and frequency (%) of the data were used to describe the study population. Unpaired student’s t-test was used to compare outcomes (survival versus death). To determine cut off point for serum lactate and CK, ROC curves were used.

Shahid Beheshti University of Medical Sciences ethics committee reviewed and approved the study protocol (IR.SBMU.RETECH.REC.1397.428) including taking oral consent. Oral consent was taken from all patients’ next of keen before recruitment into the study as we did not perform special procedures or interventions on the patients and thought verbal consent would suffice.

## Results

Of 100 patients enrolled into the study, 61 were male with a mean age of 34.01 ± 14.65 years and 39 were female with a mean age of 40.6 ± 7.49 years. Sixty-five patients survived (Table 1). Mean hospitalization period was 2.69 ± 2.12 h (range; 30 min to 5.5 h) and 94.7 ± 40.4 h in non-survivors and survivors, respectively. The most common cause of poisoning was drug overdose in 78 patients.

**Table 1:** Survivors and non-survivors based on the type of poisoning

Type of Poisoning	Total	Survivors	Non-survivors
Multidrug poisoning	28	20	8
Benzodiazepine	19	11	8
Aluminum phosphide	7	2	5
Methadone	6	4	2
Tramadol	6	4	2
Ethanol	6	3	3
Cannabis	6	5	1
SSRIs	5	2	3
Amphetamine	4	4	0
Opium	4	3	1
Beta blockers	2	1	1
Snake bite	2	2	0
Organophosphate	1	1	0
Rodenticides (anticoagulants)	1	1	0
Depilating agents	1	1	0
Inert gas poisoning	1	1	0
Hydrocarbons	1	0	1
Total	100	65	35

**Fig. 1:**
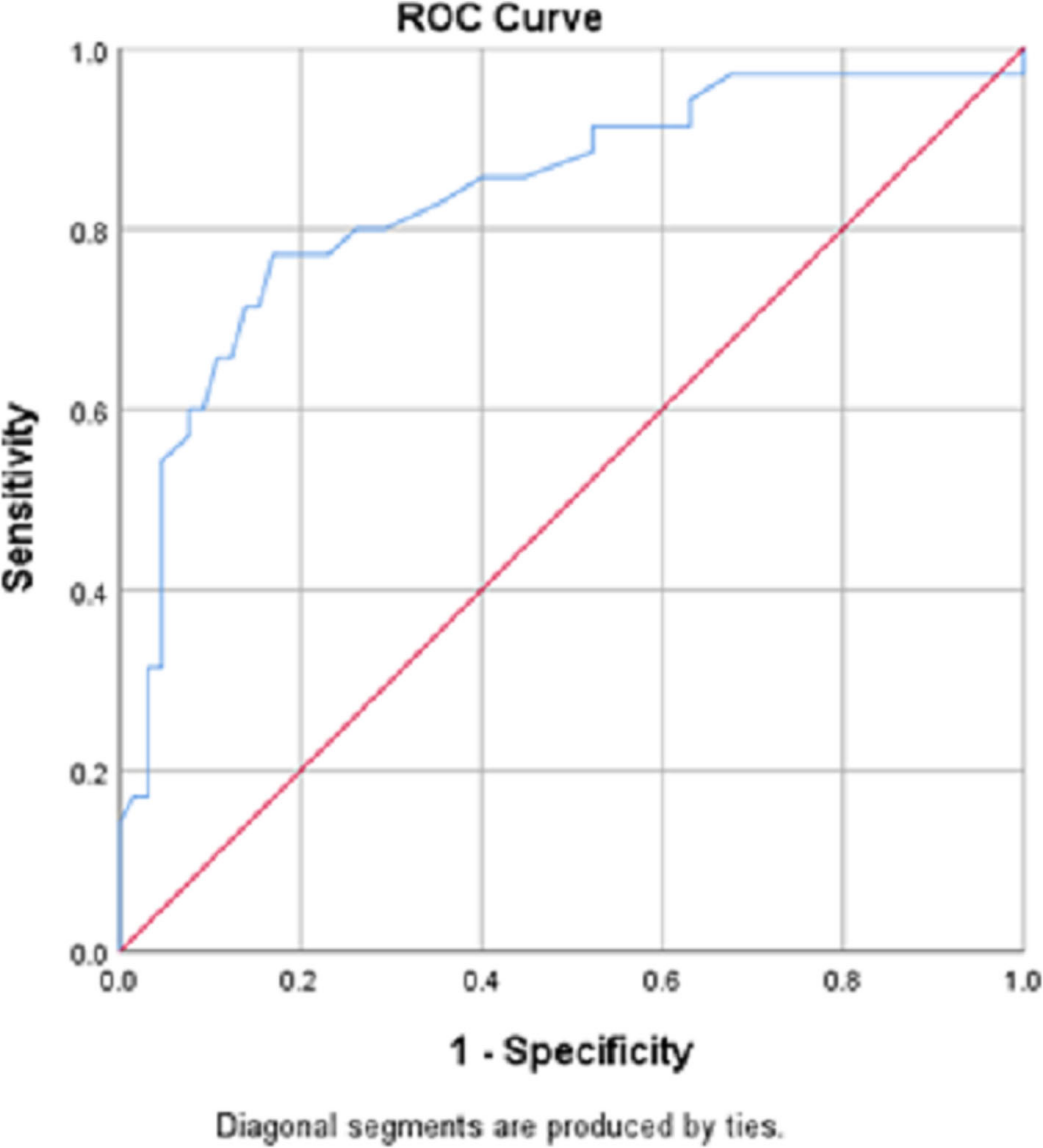
ROC curve for serum Lactate level in dead patients

**Fig. 2:**
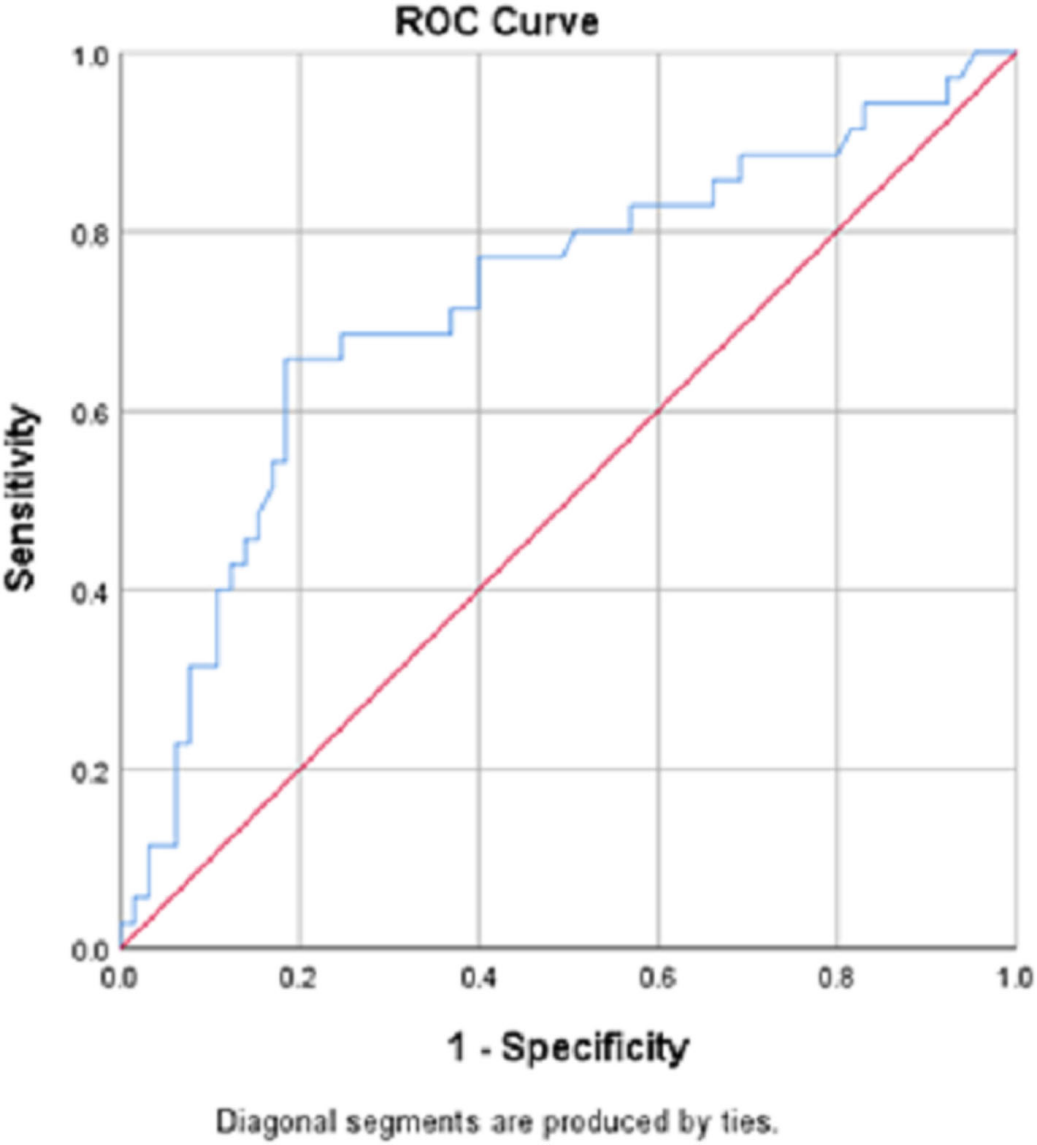
ROC curve for serum CK level in dead patient

Basic characteristics and on-presentation vitals as well as biochemistry lab tests, liver function tests, and coagulation tests were similar between the two groups of survivors and non-survivors. Treatments given (intubation, inotropes, etc) were also similar between the groups. Mean serum lactate and CK were 38.1 ± 2.4 (range; 11 to 69) mg/dL and 760.0 ± 165.3 (range; 57 to 3420) U/L in non-survivors versus 22.3 ± 0.7 (range; 12 to 58) mg/dL and 224.1 ± 32.8 (range; 33 to 1600) U/L in survivors (Ps were < 0.001 and 0.003), respectively. Spearman correlation of the study variables is shown in Table 2. Roc curves shown in Figs. 1 and 2 were used to obtain cut-offs at which the prognosis could best be determined. AUC was 0.859 (cut-off point of 26.50 ± 0.045 with sensitivity of 78% and specificity of 77%) and 0.735 (cut-off point of 169.50 ± 0.056 with sensitivity of 74% and specificity of 62%) for lactate and CK, respectively.

**Table 2:** Survey of Spearman’s correlation between study variables

	CK	Lactate	Duration of hospitalization	Consequences	Sex
CK	1.000	*0.294	**0.297	**0.348	−0.159
Lactate	0.294*	1	0.133	**0.537	−0.148
Duration of hospitalization	0.297**	0.133	1	**0.503	−0.005
Consequences	0.384**	**0.537	^**^0.503	1	0.015
Sex	−0.159	−0.148	−0.005	0.015	1

## Discussion

The prognostic and diagnostic utility of a serum lactate concentration in the initial evaluation of drug overdose is historically controversial. Lactate concentration is a useful prognostic indicator for mortality in both medical/surgical patients and undifferentiated ICU patients. Current guidelines for the initial approach to management of the patient with a drug overdose do not include routine evaluation of serum for a lactate concentration. However, lactate concentration is an established prognostic marker for the evaluation of patients with elevated anion gap metabolic acidosis, selected drug overdoses (metformin and acetaminophen), selected chronic drug toxicities (stadivudine), and chemical poisoning (aluminum phosphide and cyanide) [8–10].

As the report of national drug and poisoning information center of Iran provided, 60% of all contacts per year are related to poisoning [11]. Lactic acidosis is the condition where lactate concentration increase instantly to more than 5 mmol/dL. Type A lactic acidosis occurs in oxygen distribution dysfunction due to hypotension or cyanosis [11]. Type B lactic acidosis occurs in sepsis, liver dysfunction, diabetes, and drugs such as biguanides, acetaminophen, and sorbitol [6, 9].

Creatine kinase supplies energy in body organs with different types in brain (CK1), myocardium (CK2), and muscle (CK3) whose change is considered to be due to organ damages [5]. Usually, existence of CK in blood defines the organ injuries including myocardial infarctions, rhabdomyolysis, autoimmune myositis, and kidney injuries [7].

Our results are in accordance with those reported by Lee et al. in paraquat-poisoned patients in early stages of poisoning [12] although they did not evaluate CK level. Manini and colleagues evaluated 50 cases and 100 controls among acutely-poisoned patients and determined that lactate was an excellent prognostic factor in poisoned patients [8]. They declared that using ROC analysis, initial venous lactate concentrations obtained in the ED had outstanding diagnostic test characteristics. By maximizing the sum of sensitivity and specificity, selection of optimal integer cut points for lactate concentration occurred at 3.0 (27 mg/dL) and 5.0 mmol/L [8] which is very close to our results (the most sensitive cutoff point of 26.5 mg/dL).

Talbot and assistants defined cryptic shock as condition of shock in which blood pressure is normal but lactate is increased. They mentioned that hypoxia or toxins could destroy ATP since phosphorylation chain accelerates causing in accumulation of pyruvate and increased in Lactate level [13]. However, in a study on carbon monoxide-poisoned patients, although it was shown that serum lactate increases at the beginning of CO poisoning and this increase is compatible with patients’ blood CO level and severity of neurologic symptoms, there was no statistically significant difference in neurological symptoms in study groups. This study concluded that serum lactate was not a beneficial criterion for intoxication determining (Spearman’s test r = 0.3) [14] that is not in line with our study. Mozaffari et al. also mentioned that serum lactate was higher in patients who had died in toxicology ICU although their main focus was on ventilator-associated pneumonia and no direct correlation had been checked [15].

We also evaluated serum CK in accordance with serum lactate between survivors and non-survivors and tried to set a specific cut-off point to simplify evaluation of clinical condition of each patient and accelerate medical actions. Finally, lactate and CK level were higher in non-survivors with an acceptable cut-off point set to differentiate between these two group. It seems that Lactate and CK can be used in determination of prognosis in acute poisonings as Manini and colleagues had previously claimed [8].

## Conclusions

Regarding acceptable results and easily available tests that are sensitive enough and increase quickly after the poisoning injury, serum lactate and CK can be used as potential prognostic factors in acutely poisoned patients. This can help us better manage these patients in over-crowded poisoning wards with limited ICU beds.

## Data Availability

The data that support the findings of this study are available from the corresponding author upon reasonable request.

## Abbreviations

ICU: Intensive care unit
CK: Creatine kinase
CO: Carbon monoxide
ED: Emergency department
AUC: Area under curve
